# STAKEHOLDER ENGAGEMENT IN THE DESIGN OF A FALL RISK PREDICTION SYSTEM FOR SOCIALLY VULNERABLE OLDER ADULTS

**DOI:** 10.1093/geroni/igac059.2175

**Published:** 2022-12-20

**Authors:** George Demiris, Nancy Hodgson, Therese Richmond, Marjorie Skubic, Sean Harrison, Justine Sefcik, Sungho Oh, HanNah Cho

**Affiliations:** University of Pennsylvania, Philadelphia, Pennsylvania, United States; University of Pennsylvania, Philadelphia, Pennsylvania, United States; University of Pennsylvania, Philadelphia, Pennsylvania, United States; University of Missouri, Columbia, Missouri, United States; University of Pennsylvania, Philadelphia, Pennsylvania, United States; Drexel University, Philadelphia, Pennsylvania, United States; University of Pennsylvania, Philadelphia, Pennsylvania, United States; University of Pennsylvania, Philadelphia, Pennsylvania, United States

## Abstract

Falls and fall-related injuries are significant public health issues for adults 65 years of age and older. Fall-related injuries are among the most expensive medical conditions. Mild cognitive impairment (MCI) and housing conditions are each independent risk factors for multiple falls among older adults and there is increasing evidence of the adverse effect of MCI on balance, stride, gait speed and increasing fall risk. We developed an innovative technology-supported intervention called Sense4Safety to 1) identify escalating risk for falls through real-time in-home passive sensor monitoring (using depth sensors); 2) employ machine learning to inform individualized alerts for fall risk; and 3) link ‘at risk’ socially vulnerable older adults with a tele-coach who guides them in implementing evidence-based individualized plans to reduce fall-risk. The purpose of this study was a) to examine preferences and attitudes of low-income older adults towards an in-home sensor-based system to inform fall prevention strategies; and b) solicit feedback from and validate the intervention protocols by clinical experts in fall prevention, fall risk assessment and geriatrics. We conducted one hour interview sessions with a total of 10 older adults and 10 clinical experts. Sessions were transcribed and analyzed. Findings included perceived benefits and challenges, recommendations for refinement of the intervention (including educational components and different visualization approaches for sensor data) and the role of family members or trusted others in addressing fall risk. We highlight clinical and ethical implications resulting from the use of passive monitoring for socially vulnerable older adults with MCI.

